# A Transfer Learning Framework for Deep Learning-Based CT-to-Perfusion Mapping on Lung Cancer Patients

**DOI:** 10.3389/fonc.2022.883516

**Published:** 2022-07-01

**Authors:** Ge Ren, Bing Li, Sai-kit Lam, Haonan Xiao, Yu-Hua Huang, Andy Lai-yin Cheung, Yufei Lu, Ronghu Mao, Hong Ge, Feng-Ming (Spring) Kong, Wai-yin Ho, Jing Cai

**Affiliations:** ^1^Department of Health Technology and Informatics, The Hong Kong Polytechnic University, Hong Kong, Hong Kong SAR, China; ^2^Department of Radiotherapy, Affiliated Cancer Hospital of Zhengzhou University/Henan Cancer Hospital, Zhengzhou, China; ^3^Department of Clinical Oncology, Queen Mary Hospital, Hong Kong, Hong Kong SAR, China; ^4^Department of Clinical Oncology, The University of Hong Kong, Hong Kong, Hong Kong SAR, China; ^5^Department of Nuclear Medicine, Queen Mary Hospital, Hong Kong, Hong Kong SAR, China

**Keywords:** perfusion imaging, functional lung avoidance radiation therapy, deep learning, CT-to-perfusion translation, lung cancer, radiation therapy

## Abstract

**Purpose:**

Deep learning model has shown the feasibility of providing spatial lung perfusion information based on CT images. However, the performance of this method on lung cancer patients is yet to be investigated. This study aims to develop a transfer learning framework to evaluate the deep learning based CT-to-perfusion mapping method specifically on lung cancer patients.

**Methods:**

SPECT/CT perfusion scans of 33 lung cancer patients and 137 non-cancer patients were retrospectively collected from two hospitals. To adapt the deep learning model on lung cancer patients, a transfer learning framework was developed to utilize the features learned from the non-cancer patients. These images were processed to extract features from three-dimensional CT images and synthesize the corresponding CT-based perfusion images. A pre-trained model was first developed using a dataset of patients with lung diseases other than lung cancer, and subsequently fine-tuned specifically on lung cancer patients under three-fold cross-validation. A multi-level evaluation was performed between the CT-based perfusion images and ground-truth SPECT perfusion images in aspects of voxel-wise correlation using Spearman’s correlation coefficient (R), function-wise similarity using Dice Similarity Coefficient (DSC), and lobe-wise agreement using mean perfusion value for each lobe of the lungs.

**Results:**

The fine-tuned model yielded a high voxel-wise correlation (0.8142 ± 0.0669) and outperformed the pre-trained model by approximately 8%. Evaluation of function-wise similarity indicated an average DSC value of 0.8112 ± 0.0484 (range: 0.6460-0.8984) for high-functional lungs and 0.8137 ± 0.0414 (range: 0.6743-0.8902) for low-functional lungs. Among the 33 lung cancer patients, high DSC values of greater than 0.7 were achieved for high functional volumes in 32 patients and low functional volumes in all patients. The correlations of the mean perfusion value on the left upper lobe, left lower lobe, right upper lobe, right middle lobe, and right lower lobe were 0.7314, 0.7134, 0.5108, 0.4765, and 0.7618, respectively.

**Conclusion:**

For lung cancer patients, the CT-based perfusion images synthesized by the transfer learning framework indicated a strong voxel-wise correlation and function-wise similarity with the SPECT perfusion images. This suggests the great potential of the deep learning method in providing regional-based functional information for functional lung avoidance radiation therapy.

## Introduction

Functional Lung Avoidance Radiation Therapy (FLART) is an emerging technique that selectively avoids excessive dose delivery to the high functional lung volumes, while favoring dose deposition in the low functional lung volumes based on the information obtained from pulmonary function imaging (1–3). Currently, there are three ongoing clinical trials in the United States (NCT02528942, NCT02308709, and NCT02843568) investigating the clinical efficacy of FLART. In addition, Matuszak et al. found that the mean dose in the high functional region decreased from 12.6 ± 4.9 Gy to 9.9 ± 4.4 Gy (4). Waxweiler et al. observed an average decrease of the mean dose to the functional lung by 2.8 Gy in FLART planning (5). Yamamoto et al. reported a 5.0% decrease in the dose of the FLART planning (6). This approach holds great promise to increase post-treatment perfusion in low-dose regions and minimize radiation-induced lung injury (7, 8).

The implementation of FLART relies on lung functional images to provide information on regional lung function for guiding the treatment planning process. A number of methods have been proposed for lung function imaging, which can be broadly divided into two categories: contrast agent-based imaging methods and deformable image registration (DIR) based methods. Contrast agent-based imaging reveals lung function by using different imaging contrast agents, examples including single-photon emission computed tomography (SPECT) with Tc-99m-labelled macro aggregated albumin (MAA) (4, 7), positron emission tomography (PET) with Ga-68 (9), magnetic resonance imaging (MRI) with hyperpolarized gas (Helium-3 or Xenon-129) (10, 11), and a variety of contrast-enhanced MRI (12–14) or CT (15). On the other hand, DIR-based methods compute surrogates of regional pulmonary function from lung four-dimensional computed tomography (4D-CT) images or breath-hold CT (BHCT) image pairs through DIR algorithms and sophisticated image mathematical metrics (16–19).

Nevertheless, these current methods suffer from numerous drawbacks, impeding the widespread application of FLART in the clinic. For example, SPECT function imaging commonly offers a limited spatial resolution and incurs focal radio aerosol clumping artifacts. PET imaging requires a long imaging time and incurs inevitable image noise. Besides, both SPECT and PET imaging requires contrast agents that may release additional ionizing radiation to patients. Hyperpolarized gas MRI (HP-MRI) is free of ionizing radiation; however, it requires precious noble gases and additional equipment for hyperpolarization. On the other hand, DIR-based function imaging is error-prone due to the deficiencies of the current DIR algorithms. These limitations have restricted their widespread application in clinic (17). In general, these function imaging modalities are of low accessibility in the radiation oncology department for the patients (17, 20).

Confronted with these limitations, the deep learning-based CT-to-perfusion mapping (CTPM) method was proposed in our previous study (21). This method synthesizes lung functional images based on the texture information provided by anatomic CT images. We previously demonstrated that the CT-based perfusion images generated by the CTPM method achieved a moderate-to-high approximation as compared with SPECT perfusion images in patients with different lung diseases (22, 23). Perfusion SPECT is one of the primarily diagnostic tools for pulmonary embolism, but not for lung cancer patients. In this study, we collected a cohort of lung cancer patients with 3D SPECT perfusion images. In the hope of paving the way towards FLART application in the future, we aimed to develop a transfer learning framework to evaluate the performance of the CTPM method specifically in lung cancer patients by using multi-level evaluations (voxel-wise correlation, function-wise similarity, and lobe-wise agreement).

## Method

### Datasets and Image Acquisition

In this study, two datasets of SPECT/CT perfusion images were retrospectively collected from two hospitals. The first dataset (n=33, lung cancer dataset) was built using SPECT/CT images collected from Hong Kong Queen Mary Hospital (n=14, Institution A) and Henan Cancer Hospital (n=19, Institution B). All patients in this dataset were diagnosed with lung cancer in clinical diagnosis and the SPECT/CT scans were performed before treatment. The second dataset (n=137, non-cancer dataset) was collected from institution A, which includes different types of lung diseases except lung cancer (such as pulmonary hypertension, pulmonary embolism, etc.). The patient characteristics of the two datasets are listed in **Table 1**. This study was approved by the Institutional Review Boards (IRB) of The University of Hong Kong/Hospital Authority Hong Kong West Cluster and the IRB of Henan Cancer Hospital.

**Table 1:** Patient characteristics of the two datasets.

		Lung cancer dataset		Non-cancer dataset	
		Number	Percent	Number	Percent
Sex	Male	18	54.5%	51	37.2%
	Female	15	45.5%	86	62.8%
Age	Mean ± SD	64 ± 7.6		65 ± 15.7	

SPECT/CT scans collected from Institution A were acquired with 111 MBq technetium-99m (^99m^Tc) MAA before imaging. Patients were immobilized in the supine position with normal resting breathing during image acquisition. The 3D SPECT/CT scans were acquired in 360 degrees to cover the whole lung volume under GE Discovery 670 SPECT/CT scanner (GE Healthcare, Milwaukee, WI) with a frame rate of 30 seconds per frame and a total frame number of 60. Each acquired CT image was reconstructed into 512×512 slices with 0.977×0.977 mm^2^ in-plane pixel spacing and 1.25 mm slice thickness, while each acquired SPECT image was reconstructed into a 128×128×128 matrix with 4.42×4.42×4.42 mm^3^ voxel size.

Patients from Institution B were scanned using a dual-head SPECT-CT scanner (Philips, Eindhoven, The Netherlands). A total of 185 MBq ^99m^ Tc-MAA was injected through the brachium vein of the patient. Cross−sectional images were acquired with one frame for 60 seconds per frame. Each acquired CT image was reconstructed into a 512×512 matrix with 0.977×0.977 mm^2^ pixel spacing and 3.75 mm slice thickness, and each SPECT image was reconstructed into a 128×128×60 matrix with 2.76×2.76×1 mm^3^ voxel size.

Each SPECT image was registered to the corresponding CT image. To ensure the consistency of the acquired data between different institutions, all the acquired SPECT/CT images were reconstructed into a voxel size of 1×1×1 mm^3^. All downstream evaluations were performed under this resolution.

### Transfer Learning Framework for the Generation of CT-Based Perfusion

**Figure 1:**
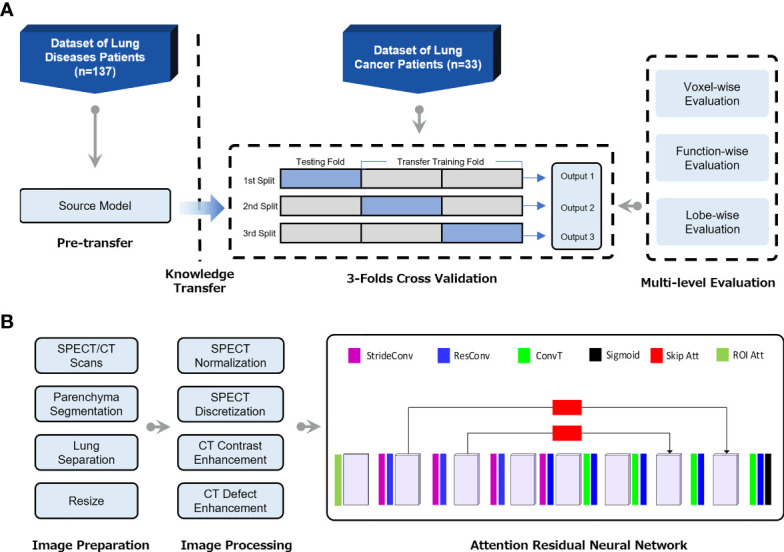
**(A)** Flow chart of the transfer learning framework for generation of CT-based perfusion images on lung cancer patients. **(B)** The pipeline of deep learning-based CT-to-perfusion mapping (CTPM) method.

To adapt the deep learning model in the lung cancer cohort, a transfer learning framework was developed to utilize the features learned from the non-cancer patients (**Figure 1A**). Specifically, the convolutional neural network (CNN) of the CTPM method was firstly trained on the non-cancer dataset to learn the fundamental mapping relation. Then the learned parameters from the non-cancer dataset were used as the initial parameters for further tunning on the lung cancer dataset. During the transfer training, three-fold cross-validation was used to make full use of the dataset. In each split, 2/3 of the lung cancer patients were used for training, with the remaining patients for testing. The outputs from three splits were combined for the subsequent evaluations.

The preprocessing procedures and CNN model were proposed in our previous study (22) and illustrated in **Figure 1B**. Briefly, the lung parenchyma region was segmented by using a pre-trained U-Net model (R231) (24), which was trained on multifarious lung CT scans. Then the left and right lungs were separated and cropped to the border of the parenchyma, followed by resampling to 128 × 64 × 64-sized matrices. The resampled CT and SPECT images were standardized using CT enhancement and SPECT standardization, respectively. In this process, the tumor regions and vessels were removed from all images and following evaluations by using thresholds of -1000 to -300 Hounsfield unit (HU). In this study, the three-dimensional attention residual neural network (ARNN) was utilized to extract features from three-dimensional CT images and synthesize the corresponding CT-based perfusion images. It was trained with the processed CT images as input and processed SPECT perfusion as the target. In the application, the trained ARNN translated the processed CT images and synthesized the corresponding perfusion images.

To ensure that the synthesized lung images were in the same shape and coordinate with the original lung CT, the output images of left/right lungs were combined and recovered to the same geometry with the pre-processed CT images, which is referred to as CT-based perfusion images. The signal intensity of a lung SPECT image is strongly affected by the patient’s condition, such as the respiratory capacity, frequency, diseases, etc. To ensure the perfusion value is comparable between patients, the SPECT perfusion was normalized to the 75th percentile value for each image, as this is close to the perfusion value of normal-functioning lung tissue (25). The CT-based perfusion images were then compared with the SPECT perfusion images *via* multi-level evaluations, including voxel-wise correlation, function-wise similarity, and lobe-wise agreement.

### Quantitative Evaluation of CT-BASED PERFUSION WITH SPECT Perfusion

#### Voxel-Wise Correlation

To evaluate the performance in terms of voxel-wise intensity correlation, the Spearman’s correlation coefficient (R) was computed between the CT-based perfusion images and the corresponding SPECT perfusion images. R is defined by the equation (1):


(1) $$R=\frac{\sum_{i=1}^{N}\left[\left(y_{i}-\bar{y}\right)\cdot \left(p_{i}-\bar{p}\right)\right]}{\sqrt{\sum_{i=1}^{N}{\left(y_{i}-\bar{y}\right)}^{2}}\sqrt{\sum_{i=1}^{N}{\left(p_{i}-\bar{p}\right)}^{2}}},$$

where $\bar{p}$ , $\bar{y}$ *p_i_*, and *y_i_* denote the average value and value at voxel *i* for the predicted and ground-truth perfusions, respectively. *N* denotes the total number of non-zero voxels.

#### Function-Wise Similarity

To evaluate the accuracy of high functional lung avoidance as well as low functional lung allowance in inverse planning, we defined the low/high functional lung volumes in both the SPECT/CT-based perfusion images for the volume overlap test. Since each perfusion image has a maximum value of 1, the threshold value of 0.66 was used to separate the low and high functional lung volumes, which were suggested in previous lung ventilation study and FLART planning (26, 27). The Dice Similarity Coefficient (DSC) was then computed to determine the similarity of the low/high functional lung volumes. DSC is defined as follows.


(2) $$DSC=\frac{2*\left|p\cap y\right|}{\left|p\right|+\left|y\right|},$$

where *p* is the low- and high-functional volume in the predicted perfusion images, and *y* is the corresponding volume in the ground-truth SPECT perfusion images. The overall concordance is inferred as the mean *DSC* value of all the testing cases.

#### Lobe-Wise Agreement

To evaluate the overlap of different lung regions, the perfusion images were segmented based on the region of lobes of the lung for further analysis. Specifically, the left upper lobe (LUL) and left lower lobe (LLL) were segmented from the left lung; the right upper lobe (RUL), right middle lobe (RML) and right lower lobe (RLL) were segmented from the right lung. The lobe segmentations were performed on the CT images using the Chest Imaging Platform in open-source software 3D Slicer (Surgical Planning Laboratory, Brigham and Women’s Hospital, Boston, Mass) (28, 29). To compare the perfusion in each lobe region, the mean perfusion value in each lobe was calculated for both SPECT/CT-based perfusion images.

### Convolution Neural Network Implementation

The CT and SPECT images were prepared and processed prior to model training and testing. The initialization of the convolutional layers was configurated using the Kaiming Uniform method (30). We implemented our network using the Pytorch 1.1 framework and coded the processing procedures in python. All the experiments were performed using a workstation with Intel Core i7-8700 @ 3.2GHz CPU, NVIDIA GTX 2080 TI GPU with 11GB memory, and 32 GB of RAM.

## Results

**Figure 2:**
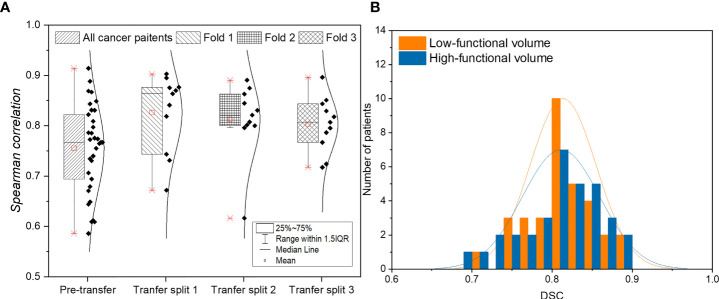
**(A)** Evaluation of voxel-wise correlation of lung cancer patients before and after applying transfer learning. **(B)** Histogram of function-wise similarity evaluation in terms of DSC for low/high functional volumes. **(A)** shows the result of voxel-wise correlation evaluation before and after transfer learning using the CTPM method. The CT-based perfusion images in the three splits after transfer learning achieved a high correlation value (R) of 0.8263 ± 0.0767, 0.8133 ± 0.0727, and 0.8032 ± 0.0537, respectively, with an average correlation value of 0.8142 ± 0.0669 for all three splits. Compared with the testing results before transfer learning (0.7554 ± 0.0875), there was a significant improvement of the average of all splits (7.78%, p = 0.0047) in the performance after fine-tuning of the model. **(B)** shows the function-wise similarity evaluation of low-functional volume (LFV) and high-functional volume (HFV) in RT treatment planning between SPECT perfusion images and CT-based perfusion images. The mean DSC for LFV and HFV were 0.8137 ± 0.0414 and 0.8112 ± 0.0484, respectively, suggesting a high similarity of both levels of functional volumes. Among the 33 lung cancer patients, a high DSC value of greater than 0.8 was achieved in 67% of patients for high-functional volume and 70% for low-functional volume; almost all the lung cancer patients (33 for low-functional volume, and 32 for high-functional volume) demonstrated a DSC value larger than 0.7.

**Figure 2A** shows the result of voxel-wise correlation evaluation before and after transfer learning using the CTPM method. The CT-based perfusion images in the three splits after transfer learning achieved a high correlation value (R) of 0.8263 ± 0.0767, 0.8133 ± 0.0727, and 0.8032 ± 0.0537, respectively, with an average correlation value of 0.8142 ± 0.0669 for all three splits. Compared with the testing results before transfer learning (0.7554 ± 0.0875), there was a significant improvement of the average of all splits (7.78%, p = 0.0047) in the performance after fine-tuning of the model.

**Figure 2B** shows the function-wise similarity evaluation of low-functional volume (LFV) and high-functional volume (HFV) in RT treatment planning between SPECT perfusion images and CT-based perfusion images. The mean DSC for LFV and HFV were 0.8137 ± 0.0414 and 0.8112 ± 0.0484, respectively, suggesting a high similarity of both levels of functional volumes. Among the 33 lung cancer patients, a high DSC value of greater than 0.8 was achieved in 67% of patients for high-functional volume and 70% for low-functional volume; almost all the lung cancer patients (33 for low-functional volume, and 32 for high-functional volume) demonstrated a DSC value larger than 0.7.

**Figure 3:**
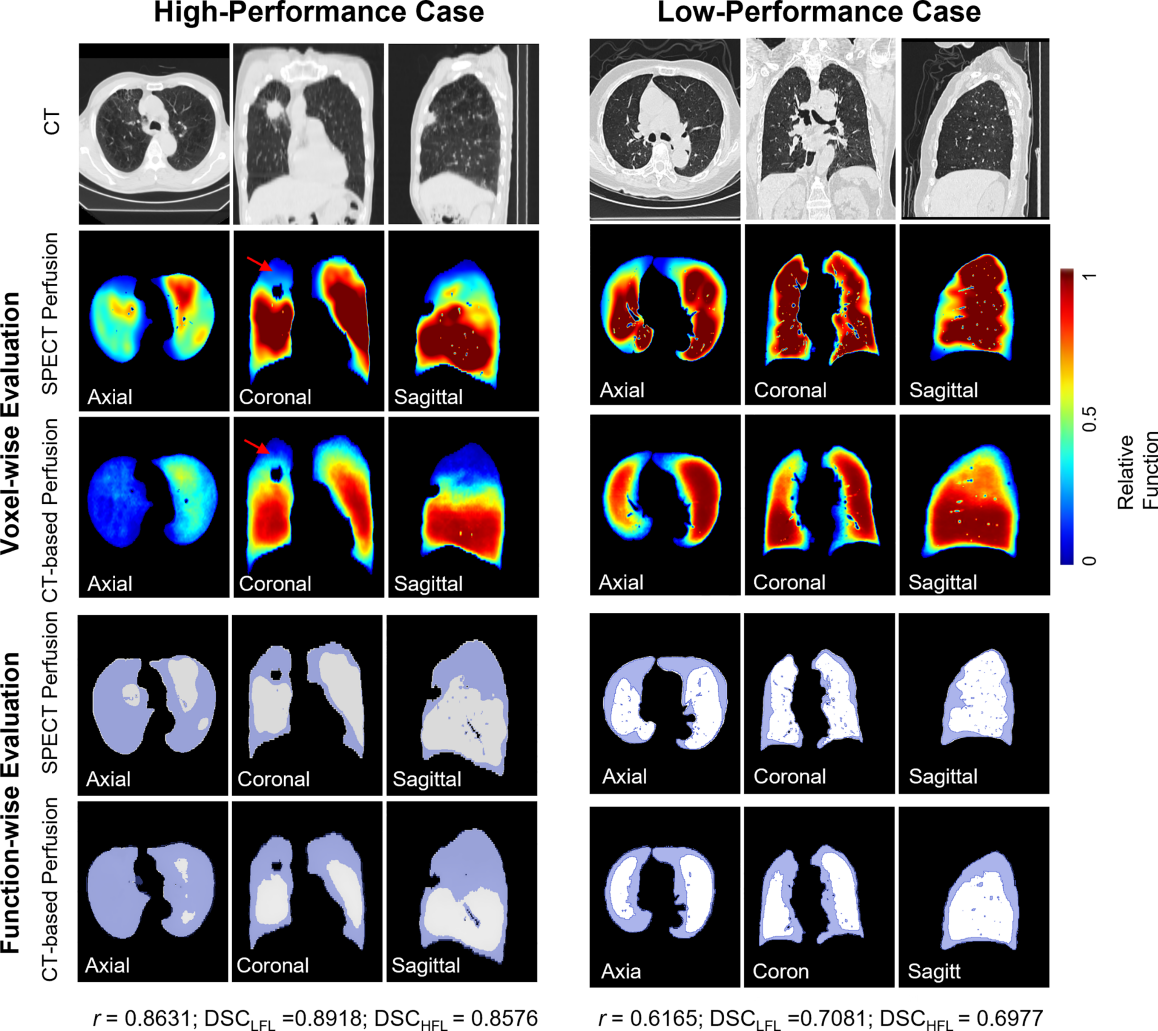
Comparison of SPECT perfusion images and CT-based perfusion images in terms of voxel-wise correlation and function-wise similarity for two representative lung cancer cases. Each case is presented in axial, coronal, and sagittal views. In the voxel-wise evaluation, the red arrow indicates the main low functional regions. In function-wise evaluation, the blue contour indicates the low functional volume for treatment planning, while the white contour indicates the high functional volume. It shows results of voxel-wise correlation and function-wise similarity in representative lung cancer patients. For the high-performance case in the testing group, the low functional region on the right upper region was successfully predicted on the CT-based perfusion. For the low-performance case in the testing group, no apparent low functional region was observed on the synthesized and ground-truth images. For both cases, CT-based perfusion images showed similar low-functional/high-functional regions to their respective SPECT perfusion images.

**Figure 3** shows results of voxel-wise correlation and function-wise similarity in representative lung cancer patients. For the high-performance case in the testing group, the low functional region on the right upper region was successfully predicted on the CT-based perfusion. For the low-performance case in the testing group, no apparent low functional region was observed on the synthesized and ground-truth images. For both cases, CT-based perfusion images showed similar low-functional/high-functional regions to their respective SPECT perfusion images.

**Figure 4:**
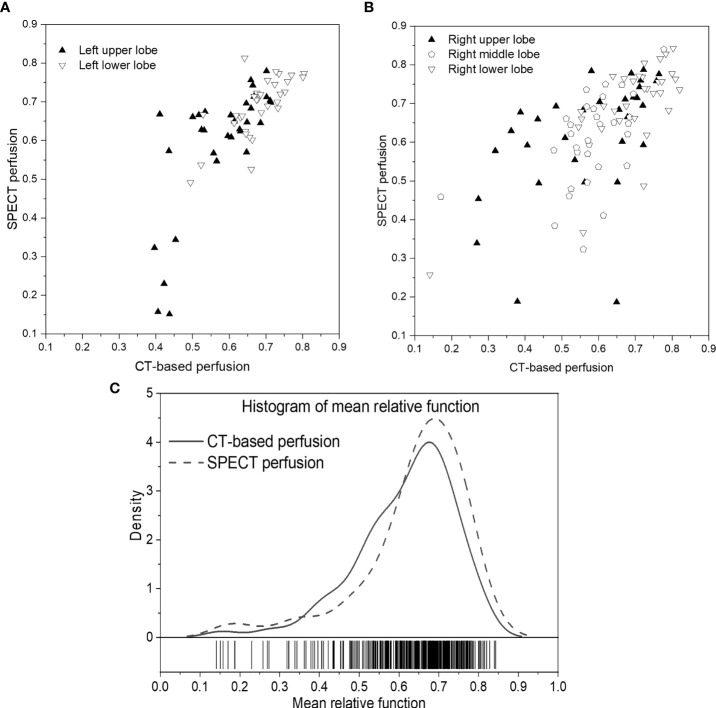
Scatter plots of the mean relative perfusion value in each lobe between SPECT perfusion images and CT-based perfusion images of the left lung **(A)** and right lung **(B)**. **(C)** Histogram of the mean relative perfusion values of all the lobes. **(A, B)** show the scatter plots of the mean value of each lobe between SPECT perfusion images and CT-based perfusion images. The correlations of the mean perfusion value on LUL, LLL, RUL, RML, and RLL were 0.7314, 0.7134, 0.5108, 0.4765, and 0.7618, respectively. The regional accuracy of CT-based perfusion on the RUL and RML was lower than the performance on other lobes. For the histogram of mean perfusion function, the histogram of CT-based perfusion images was lower than SPECT perfusion images in the range of 0 to 0.35 and 0.6 to 1, while higher on the other side.

**Figures 4A, B** show the scatter plots of the mean value of each lobe between SPECT perfusion images and CT-based perfusion images. The correlations of the mean perfusion value on LUL, LLL, RUL, RML, and RLL were 0.7314, 0.7134, 0.5108, 0.4765, and 0.7618, respectively. The regional accuracy of CT-based perfusion on the RUL and RML was lower than the performance on other lobes. For the histogram of mean perfusion function, the histogram of CT-based perfusion images was lower than SPECT perfusion images in the range of 0 to 0.35 and 0.6 to 1, while higher on the other side.

**Figure 5:**
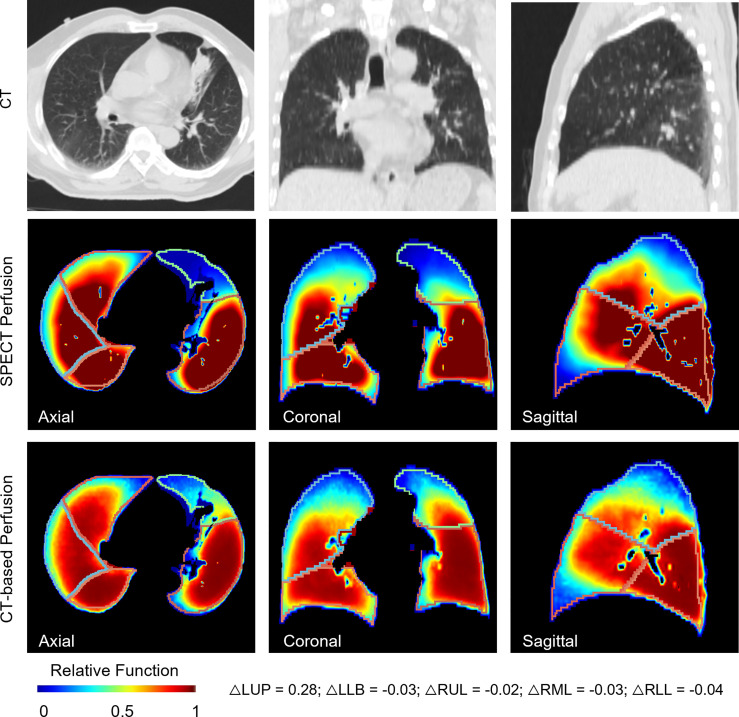
A representative case for the lobe-wise agreement between the CT-based perfusion images and the ground-truth SPECT perfusion images. It shows a representative case of lobe-wise comparison of SPECT perfusion image and CT-based perfusion image. The difference in the mean perfusion function on the LUL, LLL, RUL, RML, and RLL were 0.28, -0.03, 0.02, 0.03, and -0.04, respectively. The LUL had the lowest perfusion values on both perfusion images. For the LUL, the mean perfusion value of CT-based perfusion images was 0.44, while it was 0.15 in SPECT perfusion images. For other lung lobes, the differences between the synthesized/ground truth perfusion images were less than 10%.

**Figure 5** shows a representative case of lobe-wise comparison of SPECT perfusion image and CT-based perfusion image. The difference in the mean perfusion function on the LUL, LLL, RUL, RML, and RLL were 0.28, -0.03, 0.02, 0.03, and -0.04, respectively. The LUL had the lowest perfusion values on both perfusion images. For the LUL, the mean perfusion value of CT-based perfusion images was 0.44, while it was 0.15 in SPECT perfusion images. For other lung lobes, the differences between the synthesized/ground truth perfusion images were less than 10%.

## Discussion

This study was the first report on the evaluation of the deep learning-based CT-to-perfusion mapping method on lung cancer patients. The CT-based perfusion images were compared with ground-truth SPECT perfusion images with voxel-wise correlation, function-wise similarity, and lobe-wise agreement in 33 lung cancer patients. In our previous work, we developed and evaluated the deep learning based CTPM method in patients with various lung diseases (22). However, the performance of the CTPM method specifically on lung cancer patients is yet to be investigated due to the limited number of lung cancer patients in our previous dataset. In this study, we collected a total of 33 SPECT/CT scans of lung cancer patients from two different hospitals, and then developed the transfer learning framework to evaluate the performance of the CTPM method specifically for lung cancer patients, in the hope of paving the way towards FLART application in the future.

To increase the model generalizability, we first trained the CNN model on patients with various lung diseases other than lung cancer and directly adapted it to the lung cancer dataset. The CNN model achieved a voxel-wise correlation (R) of 0.7554 ± 0.0875 in lung cancer patients. Subsequently, we used a transfer learning strategy to adapt the pre-trained model to the lung cancer dataset. It was observed that the correlation was approximately 8% higher (p = 0.0047) to 0.8142 ± 0.0669 after applying the transfer learning (**Figure 2**). Transfer learning can improve the performance of the target domain by transferring the knowledge contained in different but related source domains (31). For the task of functional image synthesis, this improvement could be explained by the subject uniformity in the lung cancer dataset. Decreased lung function is caused by various mechanisms (32). For example, a complete defect can be induced by chronic obstructive pulmonary disease or other unrecoverable diseases. Lung cancer can also cause large vessel compression and alter the blood supply within the regional lung (33). In the lung cancer dataset, there are more low perfusion regions induced by tumor compression, which increases the uniformity of the low function region. For these functional defects induced by pulmonary vessel compression, tumor regression from RT may lead to regional lung reperfusion because of the relief of obstructions (34). For the future implementation of FLART, it is necessary to fine-tune the model on the lung cancer dataset to achieve better performance.

With regard to the function-wise similarity of the CT-based perfusion images, the low/high functional volumes showed almost the same level of high similarity (~0.8). In the qualitative comparison (**Figure 3**), we noticed two patterns of distributions of low functional volume (LFV): the first case had the LFV located at the corner of the lung (corner type); the second LFV was located at the peripheral region of the whole lung volume (peripheral type). The LFV of peripheral type could be attributed to its distance to the pulmonary arteries. Therefore, no significant dose-spare would be expected for this type in the FLART planning. This indicates that the benefit of generated CT-based perfusion for the surrounding type is limited. Based on this indication, a further step is needed to identify the distribution pattern of low functional regions prior to FLART implementation in the clinic.

For the lobe-wise agreement, the correlation coefficients of the mean perfusion value in the LUL (0.7314), LLL (0.7139), and RLL (0.7618) were significantly higher than those in the RUL (0.5108), RML (0.4765) regions of the lung. A possible explanation could be related to the fact that the horizontal fissure separating the RUL and RML has increased the perfusion complexity of this region. As compared with the right lung, the extra horizontal fissure makes the perfusion condition on the RUL/RML region more complex, leading to relatively large uncertainty in these regions. In the future, a vessel-based analysis will be needed to explore further the effects caused by vessel differences between the left/right lungs and increase the prediction accuracy in these regions.

**Figure 6:**
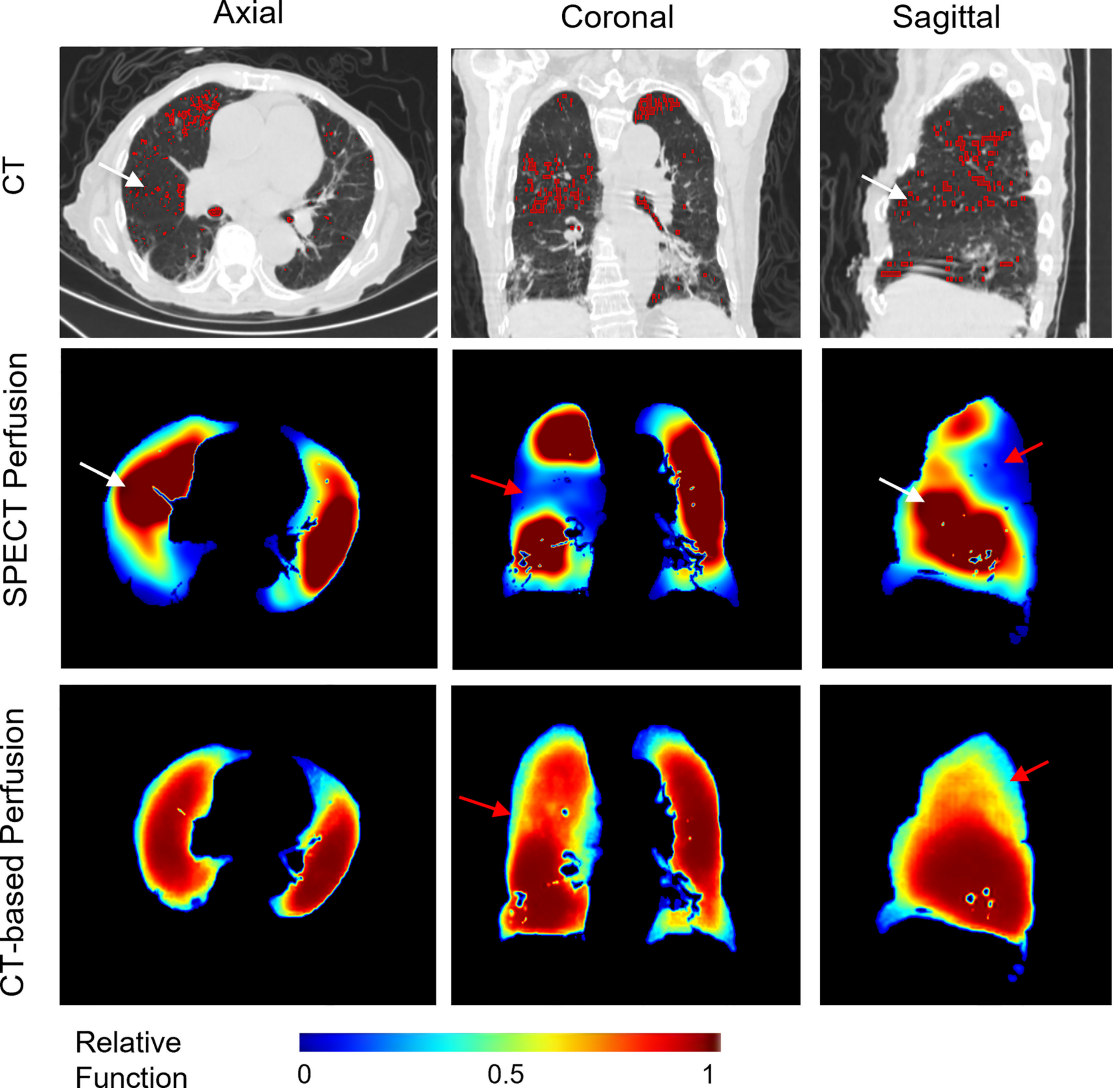
A representative case with relatively low perfusion prediction on CT-based perfusion images. The red contours in the CT images indicate the low intensity regions (<-900 HU). The red arrows indicate the main mismatched low functional regions. The white arrows indicate the inconsistent regions between the low intensity regions of the CT images and the SPECT perfusion images.

Apart from this, we also observed some cases with mismatched defect regions. As shown in **Figure 6**, the representative case has a correlation value of 0.6601 and 0.7859 for the right and left lungs, respectively. Most of the low perfusion region on the right lung (red arrow) was predicted as relatively high functional regions on synthesized CT-based perfusion. To a degree, this could be partly ascribed to the observed variations of CT-to-SPECT perfusion relationship between imaging views. In **Figure 6**, for instance, the representative case presents a consistent location between the low intensity regions (<-900 HU) within the right lung on the coronal view of the CT image (as indicated by the red regions in the first row of **Figure 6**) and the corresponding low perfusion regions on the ground-truth SPECT perfusion image (as indicated by the blue-shaded regions in the second row of **Figure 6**); while this consistency diminishes in some regions of the sagittal and axial views (as indicated by the white arrows in **Figure 6**). In this model, the regional inconsistency might have impeded accurate prediction of lung perfusion information from CT to SPECT images, and led to a “trade-off” predicting strategy of the deep learning model. This trade-off can also be observed in the histogram distribution of the mean perfusion values of all the lobes: the CTPM method tends to yield lower predicted values than the ground-truth in the perfusion value range from 0 to 0.35 and 0.6 to 1, while higher in the range from 0.35 to 0.6. When encountering uncertainties, the ARNN model trended to output median values to minimize the difference. This mismatch may degrade the accuracy of FLART treatment planning. To further improve the model performance on these uncertainty regions, The texture information of these mismatched regions should be further investigated (35).

In this study, we also compared the two groups of patients collected from two medical institutions. The performance on patients from institution A was significantly higher than that on patients from institution B in terms of correlation (10% higher, p = 0.0016), DSC of high functional volume (5% higher, p = 0.0033) and low functional volume (5% higher, p = 0.0027). These discrepancies could be explained by the different tumor sizes of these two groups of patients. There is a significant difference between the diameter of the tumors from the two institutions (p = 0.0006), with average diameter sizes of 40 ± 19.5 mm and 15 ± 15.6 mm, respectively. All the lung cancer patients from cohort A had tumor sizes larger than 20 mm, while only 6 of 14 patients from cohort B had comparable tumor sizes. The large tumor volume may have changed the blood supply and generated more significant lung functional volumes. In the future application of FLART, the effects of the tumor size should also be considered for lung function prediction.

There are also several limitations to this study. First, due to the limitation of GPU memory and the size of the training dataset, each lung is divided into left and right parts before inputting to the CNN network for model development. This may limit the cross-lung quantitative comparisons between left and right lungs from the same patient. The neural network still needs optimization in the coming study. Second, the performance of the CT-based perfusion images in treatment planning is still unknown. As such, dosimetry evaluation is still needed for potential dosimetry benefits of the CTPM method (36).

## Conclusion

In this study, we, for the first time, quantitatively developed a transfer learning framework to evaluate the deep learning based CT-to-perfusion mapping method specifically on 33 lung cancer patients at multiple levels, and achieved high correlations between the CT-based perfusion images and the ground-truth SPECT perfusion images. These findings suggested the use of CT-based perfusion images for high functional lung avoidance as well as low functional lung allowance in RT inverse planning, holding great promise in providing regional-based functional information for FLART in the future.

## Data Availability Statement

The data analyzed in this study is subject to the following licenses/restrictions: The patient images were collected from hospitals retrospectively. Requests to access these datasets should be directed to gary-ge.ren@polyu.edu.hk.

## Ethics Statement

The studies involving human participants were reviewed and approved by Institutional Review Boards (IRB) of The University of Hong Kong/Hospital Authority Hong Kong West Cluster and the IRB of Henan Cancer Hospital. The patients/participants provided their written informed consent to participate in this study.

## Author Contributions

JC and GR conceived of the presented idea. BL, HX, and YH verified the analytical methods. S-KL helped to revise the manuscript. AC, YL, RM, HG, F-MK, and WH helped to collect the data. All authors contributed to the article and approved the submitted version.

## Funding

This research was partly supported by research grants of General Research Fund (GRF 15103520), the University Grants Committee, and Health and Medical Research Fund (HMRF 07183266), the Food and Health Bureau, The Government of the Hong Kong Special Administrative Region.

## Conflict of Interest

The authors declare that the research was conducted in the absence of any commercial or financial relationships that could be construed as a potential conflict of interest.

## Publisher’s Note

All claims expressed in this article are solely those of the authors and do not necessarily represent those of their affiliated organizations, or those of the publisher, the editors and the reviewers. Any product that may be evaluated in this article, or claim that may be made by its manufacturer, is not guaranteed or endorsed by the publisher.
